# Alterations in coagulation profile of patients with periprosthetic joint infections

**DOI:** 10.1007/s00264-025-06537-w

**Published:** 2025-04-29

**Authors:** Augusto Carlos Maciel Saraiva, Alan de Paula Mozella, Hugo Alexandre de Araujo Barros Cobra, Osamu de Sandes Kimura, João Antonio Matheus Guimarães, Helton Defino, Ana Carolina Leal

**Affiliations:** 1 Orthopaedic Department, Federal University of Rio de Janeiro, Rio de Janeiro, Brazil; 2 Center for Surgery of Knee, National Institute of Traumatology and Orthopaedics, Rio de Janeiro, Brazil; 3 Medical Sciences University, State University of Rio de Janeiro (UERJ), Rio de Janeiro, Brasil; 4 Center for Surgery of Hip, National Institute of Traumatology and Orthopaedics, Rio de Janeiro, Brazil; 5 Teaching and Research Division, National Institute of Traumatology and Orthopaedics, Avenida Brasil, 500, Rio de Janeiro, RJ 20940-070 Brasil; 6 Department of Orthopaedics and Anaesthesiology, Ribeirão Preto Medical School, University of São Paulo, São Paulo, Brazil

**Keywords:** Periprosthetic joint infection, Coagulation, Biomarker, Haemostasis

## Abstract

**Purpose:**

This study aims to evaluate changes in the coagulation profile of patients with knee periprosthetic infections (PJI) and determine its diagnostic value in this complication.

**Methods:**

A prospective study was conducted with 112 patients who underwent revision surgery for total knee arthroplasty in a single tertiary hospital between January 2021 and December 2022.

**Results:**

51 patients were diagnosed with PJI. D-dimer (*p* = 0.001), fibrinogen (*p* = 0.0007), platelets (0.01), and international normalized ratio (*p* = 0.01) were significantly higher in patients with PJI.

**Conclusions:**

Patients with PJI display altered coagulation profile. The evaluation of coagulation-related markers has limited value for diagnosing PJI. Further studies are needed to understand the impact of such alterations on patients’ outcomes.

## Introduction

Periprosthetic joint infection (PJI) is one of the most severe complications associated with arthroplasties. It is estimated that 0.5 to 1.9% of patients who undergo arthroplasty will develop this complication. In some series, PJI has already been reported as the leading cause of TKA revision surgery [1–3]. This number tends to rise in the future since it is estimated that the increase in the number of arthroplasties will be accompanied by a proportional increase in the occurrence of this complication [4].

The diagnosis of PJI is still a challenge for surgeons. In recent years, the medical and scientific communities have tried identifying new biomarkers and developing algorithms that help diagnose this infection [5, 6]. The main biomarkers applied in diagnosing PJI evaluate two main aspects of the infection: the presence of bacteria and the immune response.

However, recently, two molecules involved in the coagulation process, D-dimer and fibrinogen, have been investigated and were found to increase in patients with PJI compared to patients with aseptic failure, showing promising results when used for the diagnosis of this complication [7, 8]. Accordingly, several studies have investigated the relationship between the immune and coagulation systems in infectious processes, showing that the coagulation system can be activated by different pathogens, such as viruses or bacteria, acting together with the immune system to fight the infection [9, 10]. Thus, since the evaluation of the coagulation profile is part of the routine exams to prepare patients for surgery, this study aimed to identify changes in the coagulation profile of patients with PJI and to determine its diagnostic performance for PJI diagnosis.

## Materials and methods

### Study design and patient cohort

This prospective cohort study was conducted in a tertiary health-care center specialized in high-complexity orthopaedic surgery and was approved by the Human Ethics Committee of the *Instituto Nacional de Traumatologia e Ortopedia Jamil Haddad*.

**Fig. 1:**
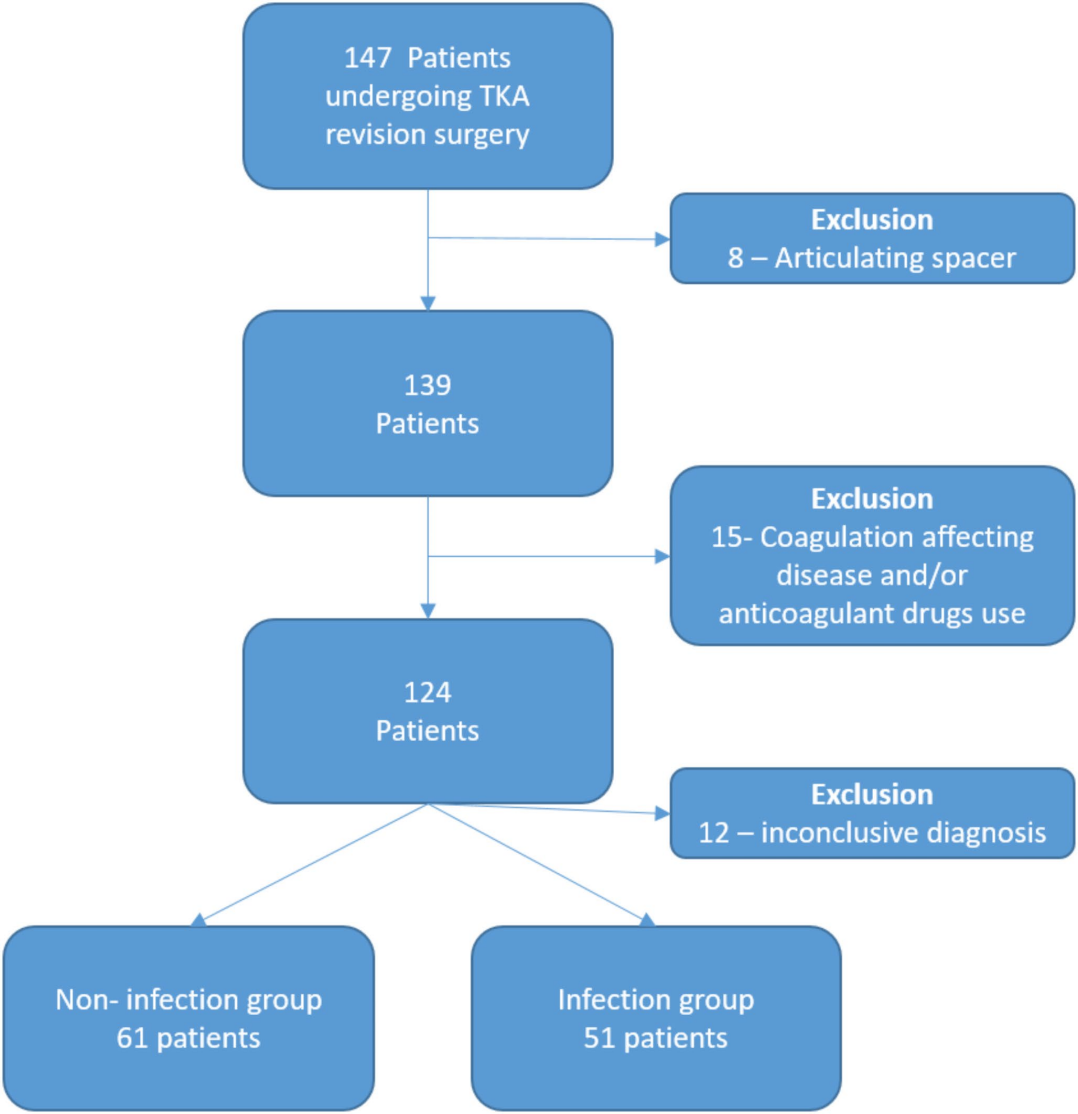
Flow chart of the study population

Patients submitted to a knee arthroplasty revision surgery between January 2021 and December 2022 were included. All patients provided informed consent for this study. Exclusion criteria included acute PJI, patients with coagulations disorders, chronic diseases known to affect coagulation (cancer, renal disease) or in use of anticoagulant drugs, and lack of appropriate clinical and laboratory data for PJI diagnosis. Chronic PJI was diagnosed according to the criteria of the Second International Consensus Meeting on Musculoskeletal Infection (2018) [6]. Participants with at least one of the major criteria or a score of 6 or more composed the PJI group. Patients with a score < 3 were diagnosed as uninfected and composed the non-PJI group. Patients with inconclusive diagnoses, i.e., scoring 3–5 points, were excluded (Fig. 1).

Synovial fluid was obtained during the revision procedure prior to the arthrotomy. One to three milliliters were drawn into vials for aerobic and anaerobic cultures (Becton, Dickinson and Company, New Jersey, USA). Culture dishes were held for 14 days and discarded after turning positive results according to a laboratory protocol. In addition to that, five to seven periprosthetic tissue samples were also harvested and sent for microbiological analyses. Histopathological analyses were also performed on periprosthetic membranes, which were classified according to the Morawietz et al. criteria [11].

One milliliter of synovial fluid was transferred to EDTA-containing tubes and used for leukocyte count and percentage of polymorphonuclear cells by flow cytometry using the automated haematology analyzer Cell Dyn 3700 SL (Abbott Diagnostics, Abbott Diagnostics, Illinois, USA).

Preoperative CRP, ESR, fibrinogen, D-dimer, platelets count, prothrombin time/international normalized ratio (INR), and activated partial thromboplastin time (aPTT) were recorded.

Data distribution was analyzed using the D’Agostino-Pearson normality test. The Mann-Whitney U test was used to compare biomarker levels between groups. Categorical variables were compared using the χ2 test. ROC curves were constructed by calculating AUC, sensitivity, and specificity at different cut-off levels. The optimum thresholds were calculated using the Youden statistic. A significance level of 0.05 was set for all tests. These statistical analyses were performed using MedCalc software version 21 (USA).

## Results

The study cohort consisted of 112 patients, of whom 51 had a confirmed diagnosis of PJI. The PJI and non-PJI groups showed no significant differences in terms of sex, age, or BMI. Demographic data and baseline characteristics of infected and non-infected cohorts are presented in Table 1.

**Table 1:** Characteristics of study population

Variable	Non-infection (*n* = 61)	Infection (*n* = 51)	*P*-value
Age (years)	71 ± 7.3	70 ± 8.6	0.30
Sex			
Male	18	20	0,31
Female	43	31	
BMI (kg/m^2^)	30.4 ± 5.2	29.4 ± 4.9	0,37
CRP	1.9 ± 7.1	5.1 ± 6.6	0.0001
ESR	41.6 ± 26.1	63.1 ± 30.5	0.0006
SF leukocytes	713.9 ± 805	31,024 ± 60,454	< 0.0001
SF % polymorphonuclear cells	21.1 ± 14.7	51.6 ± 32.3	< 0.0001

BMI: body mass index; CRP: C-reactive protein; ESR: erythrocyte sedimentation rate; SF: synovial fluid

Of the participants in the infection group, eleven had a fistula. Two cases had a negative microbiological culture. Fourty nine patients showed growth of microorganisms in the microbial culture of two or more samples. Seventeen participants had polymicrobial infections. The most frequently identified pathogen was coagulase-negative Staphylococcus. The main gram-negative bacteria identified were: *Acinetobacter baumanii*,* Serratia marscescens*,* Pseudomonas aeruginosa*,* Klebisiella pneumoniae and Enterobacter coclae*. Table 2 describes the bacteria identified in the microbiological cultures of patients with PJI and the frequency with which they were identified.

**Table 2:** Microbiological profile of patients in infection group

Microorganism	Frequency
*Staphylococcus aureus*	17
*Staphylococcus* coagulase negative	18
*Enterococcus faecalis*	7
*Streptococcus spp*	1
Bacillus spp	1
Gram-negative	15
Candida spp	1
Polymicrobial	17
Negative culture	2

Regarding coagulation-related parameters, D-dimer, fibrinogen, platelets and INR were higher in the infected group in comparison to the non-infected onde. We did not observe differences between groups regarding aPTT (Table 3).

**Table 3:** Evaluation of coagulation markers

	Non-infection	Infection	*P*-value
D-dimer (mg/L)	1.2 ± 1.7	3.4 ± 2.7	0.01
Fibrinogen (mg/dl)	333.6 ± 105	458.5 ± 180.8	0.0007
Platelets (/mm^3^)	226,274 ± 74,648	280,465 ± 123,828	0.01
INR	1.06 ± 0.06	1.11 ± 0.10	0.01
aPTT	1.00 ± 0.14	1.04 ± 0.12	0.35

INR: International normalized ratio; aPTT: Activated partial thromboplastin time

ROC curve analyses revealed a limited diagnostic value for fibrinogen, D-dimer, platelet count, and INR. Table 4 summarizes the sensitivity, specificity, and the optimal threshold for the biomarkers evaluated.

**Table 4:** Diagnostic performance of serum biomarkers

Parameter	AUC ROC	Youden index	Threshold	Sensitivity	Specificity
CRP	0.70 [0.61–0.79]	0,408	> 2.7	50.9	89.3
CRP *	-	-	> 1	60.8	66.1
ESR	0.69 [0.59–0.78]	0.38	> 71	48.9	89.3
ESR*	-	-	> 30	82.2	42.8
D-dimer	0.66 [0.55–0.76]	0.39	> 2.5	59.5	79.5
Fibrinogen	0.73 [0.62–0,83]	0.46	> 420	60.7	85
INR	0.64 [0.54–0.72]	0.22	> 1,1	39.2	83.6
Platelets	0.63 [ 0.53–0.72]	0.29	> 264,100	47	81.9

* Evaluation considering ICM 2018 proposed threshold. CRP: C-reactive protein; ESR: erythrocyte sedimentation rate; INR: International normalized ratio

## Discussion

Our results have shown that PJI patients display an altered coagulation profile characterized by increased platelet count, fibrinogen, D-dimer and INR. PJI pathophysiology involves a complex interaction between different immune system cells, mediated by the release of several factors and cytokines. In recent years, studies have demonstrated that the activation of the immune system is related to the activation of the coagulation system and that both systems act synergistically in the fight against infections [9, 10]. Ours results corroborate this idea, as we have shown that this reciprocal activation of the immune and coagulation systems also occur in the context of PJI.

Saxena and collaborators have shown that patients undergoing revision arthroplasty due to PJI displayed elevated INR compared to patients undergoing aseptic revision arthroplasty [12]. More recently, Li and collaborators also demonstrated that patients with PJI present subclinical alterations in the coagulation cascade, with increased platelet count, fibrinogen, D-dimer, INR, and aPTT [13]. Our results corroborate the findings of such studies as we have found that PJI patients present increased D-dimer, INR, fibrinogen, and platelet count. However, we did not observe significant changes in aPTT.

The alterations reported in the present study suggest that PJI is related to alterations only in the extrinsic pathway of the coagulation cascade since we identified alterations in INR but not in aPTT. This result differs from other studies that suggest alterations in both extrinsic and intrinsic pathways [12, 13]. Such studies, however, included patients with acute or chronic PJI. Therefore, one cannot exclude the impact of surgical-trauma induced alteration on coagulation factors on the presented results.

The evaluation of the diagnostic performance of coagulation parameters for diagnosing PJI revealed that fibrinogen performed better than all coagulation parameters evaluated, including D-dimer, albeit D-dimer was included as a minor criteria on the ICM 2018 algorithm. Overall, all coagulation parameters evaluated presented a limited value for PJI diagnosis. Li and collaborators conducted a similar study with 371 patients to assess the diagnostic value of the coagulation-related molecules for PJI identification [13]. Authors have shown that aPTT displayed reduced diagnostic value compared to other parameters such as fibrinogen and D-dimer. This author also proposed a combined model for using coagulation parameters in the diagnosis of PJI, obtaining a sensitivity of 79% and a specificity of 82% [13].

Taken together, the changes observed in platelets count, fibrinogen, and INR but not in aPTT are similar to those observed in patients with cancer and infectious diseases like COVID-19 [14, 15]. Such patients usually display an increase in fibrinogen and D-dimer, moderate changes in the number of platelets, and an increase in the INR. Such changes are associated with the establishment of a condition called compensated chronic disseminated intravascular coagulation, in which the consumption of coagulation factors is gradually replaced, resulting in the absence of clinical symptoms and minimal or no changes in laboratory tests [14, 16]. Over weeks to months, such a condition can cause thromboembolic manifestations, although thrombosis may not be evident, as it occurs mainly in the microvasculature [16].

Two recent articles highlight the potential risk of these hemostatic changes in patients with PJI. The first study, carried out by Bass and colleagues, demonstrated that patients with PJI have a 2.6 times greater risk of developing thromboembolic events after arthroplasty revision surgery than patients undergoing aseptic revision [17]. Another study is a case report by Papen and collaborators in which the authors present the case of a patient with PJI caused by streptococci who presented with reactive thrombosis and recurrent arterial thrombosis. In this patient, treatment of PJI with debridement and implant retention led to the cessation of thrombosis episodes, highlighting the intimate relationship between infection and coagulation [18].

This study presents some limitations. First we evaluated only patient undergoing TKA revision surgery. Second we have not performed subgroups analyses to investigate if causative microorganism would affect coagulation changes.

## Conclusion

Our results show that PJI is associated with alterations in coagulation and suggest that coagulation-related parameters may be helpful in the diagnosis of this complication. Further studies are needed to uncover the potential implications of such changes on patients’ health and treatment outcomes.

## Data Availability

The datasets used and/or analysed during the current study are available from the corresponding author on reasonable request.
